# Does Retinal Neurodegeneration Seen in Diabetic Patients Begin in the Insulin Resistance Stage?

**DOI:** 10.4274/tjo.68888

**Published:** 2016-12-01

**Authors:** Sedat Arıkan, İsmail Erşan, Mustafa Eroğlu, Mehmet Yılmaz, Hasan Ali Tufan, Baran Gencer, Selçuk Kara, Mehmet Aşık

**Affiliations:** 1 Çanakkale Onsekiz Mart University Faculty of Medicine, Department of Ophthalmology, Çanakkale, Turkey; 2 Çanakkale Onsekiz Mart University Faculty of Medicine, Department of Endocrinology and Metabolism, Çanakkale, Turkey

**Keywords:** insulin resistance, retinal ganglion cell layer, contrast sensitivity

## Abstract

**Objectives:**

To investigate whether retinal neurodegeneration and impairment in contrast sensitivity (CS), which have been demonstrated to begin in diabetic patients before the presence of signs of diabetic retinal vasculopathy, also occur in the stage of insulin resistance.

**Materials and Methods:**

The average, minimum and sectoral (inferior, superior, inferonasal, superonasal, inferotemporal and superotemporal) thicknesses of the ganglion cell-inner plexiform layer (GCIPL) measured using optical coherence tomography were compared between an insulin-resistant group and control group in order to evaluate the presence of retinal neurodegeneration. The CS of the two groups was also compared according to the logarithmic values measured at spatial frequencies of 1.5, 3, 6, 12 and 18 cycles per degree in photopic light using functional acuity contrast test (FACT).

**Results:**

Twenty-five eyes of 25 patients with insulin resistance (insulin resistant group) and 25 eyes of 25 healthy subjects (control group) were included in this study. There were no statistically significant differences between the two groups in any of the spatial frequencies in the FACT. The mean average GCIPL thickness and mean GCIPL thickness in the inferotemporal sector were significantly less in the insulin-resistant group when compared with the control group (mean average GCIPL thicknesses in the insulin-resistant and control groups were 83.6±4.7 µm and 86.7±3.7 µm respectively, p=0.01; mean inferotemporal GCIPL thicknesses in the insulin-resistant and control groups were 83±6.0 µm and 86.7±4.6 µm respectively, p=0.02).

**Conclusion:**

Although it may not lead to functional visual impairment such as CS loss, the retinal neurodegeneration seen in diabetic patients may begin in the insulin resistance stage.

## INTRODUCTION

Diabetes mellitus continues to be an important public health problem that adversely affects quality of life through serious microvascular complications such as retinopathy, nephropathy and neuropathy. The prevalence of this disease is steadily rising; it is estimated that the total number of patients with diabetes will reach 366 million by 2030, compared to 171 million in 2000.^1^ Thus, the investigation and management of factors responsible for the development of diabetes and its complications have become particularly important in order to prevent this increase. Several clinical trials including the Diabetic Control and Complication Trial and EURODIAB Prospective Complications Study have provided clinical evidence that confirm insulin resistance as a major risk factor for the development of diabetes and diabetic retinopathy (DR).^2,3,4^ In addition to its role in the pathogenesis of DR, insulin resistance was also found to be an important factor related to the occurrence of other microvascular complications of diabetes through vascular endothelial injury.^5^

It has been proposed that impaired insulin action, which is the primary defect of diabetes, directly affects the retina and may initiate retinal dysfunction.^6^ Several clinical trials investigating retinal functions in diabetic patients without DR have revealed the neurodegenerative component of DR can begin even before the occurrence of retinal vasculopathic manifestations of diabetes.^7,8,9^ This concept has also been supported by histopathological examination. Wolter^10^ demonstrated the atrophy of ganglion cells and degeneration of the inner nuclear layer in the retinas of patients with early diabetes and reported that neuronal degeneration of the retina seen in diabetic patients may be a primary pathology leading to vascular changes. Gastinger et al.^11^ have shown the loss of retinal ganglion cells (RGCs) within the first 3 months of diabetes in mice. Abu-El-Asrar et al.^12^ suggested that RGCs are the cells most vulnerable to the increased apoptosis that occurs in diabetic retina.

Apart from histopathological studies, the decrease in the thickness of the RGC layer has been clinically demonstrated using optical coherence tomography (OCT) both in patients with type 1 diabetes and in patients with type 2 diabetes with minimal or no retinopathy.^13,14^ Although the neuroprotective effect of insulin on retinal neurons has been reported in previous studies,^15,16^ there are no studies investigating the presence of neurodegeneration in patients with insulin resistance. In ophthalmic practice, spectral-domain OCT (SD-OCT) in particular is a widely used tool for early detection of the structural changes that occur in the retinal layers and for follow-up of the disease’s progression.^17^ Unlike other SD-OCTs, high-definition (HD)-OCT enables us to assess the thicknesses of the retinal nerve fiber layer (RNFL) and ganglion cell-inner plexiform layer (GCIPL) separately.^18^ Besides that, the contrast sensitivity (CS) test has been shown to be beneficial in the detection of functional changes that may occur in the early stages of glaucoma in patients with good visual acuity.^19^

Taken together, it is reasonable to use OCT along with CS test for the structural and functional evaluation of possible early retinal neurodegeneration which is thought to arise from insulin resistance. Therefore, in this study we aimed to compare CS test results and RNFL and GCIPL thicknesses between patients with insulin resistance and healthy subjects in order to evaluate the structural and functional effects of impaired insulin sensitivity on the retina.

## MATERIALS AND METHODS

This prospective, comparative study was carried out in the Ophthalmology Department of Çanakkale Onsekiz Mart University Faculty of Medicine. After the local ethics committee approved the study protocol according to the Declaration of Helsinki for research involving human subjects, healthy subjects and patients who were diagnosed as having insulin resistance and were followed in the Endocrinology and Metabolism Departments of Çanakkale Onsekiz Mart University Faculty of Medicine were recruited for the study. Written informed consent was obtained from each patient with insulin resistance and from each healthy subject who agreed to participate in this study as a volunteer. All participants underwent a comprehensive ophthalmologic examination consisting of measurement of best corrected visual acuity (BCVA) and intraocular pressure, slit-lamp biomicroscopy and funduscopic examination.

Patients meeting one or more of the following exclusion criteria were not included in the study: history of previous ocular surgery or eye trauma; contact lens wear; corneal and conjunctival diseases; ocular inflammatory diseases; dry eye disease; diagnosis of glaucoma or ocular hypertension (intraocular pressure >22 mmHg); vascular or degenerative retinal diseases; systemic diseases which can lead to retinal or optic nerve disorders (hypertension, diabetes mellitus, multiple sclerosis, etc.); diagnosis of cataract; BCVA level greater than 0 logMAR; refractive error exceeding ±2.0 diopter as spherical equivalent (SE) value. Participants whose homeostasis model assessment of insulin resistance index (HOMA-IR) [fasting insulin (μU/mL)×fasting glucose (mmol)/22.5)] value ≥2.7 was accepted to have insulin resistance. Apart from HOMA-IR value, plasma insulin level and body mass index (BMI) value were also assessed for each patient. The participants who met the eligibility criteria were assessed by CS test using functional acuity contrast test (FACT) (OPTEC 6500 Stereo Optical Co., Chicago, IL, USA) and OCT imagining using Cirrus HD-OCT 4000 (Carl Zeiss Meditec Inc., Dublin, CA, USA) consecutively.

### Contrast Sensitivity Test

Binocular CS of participants was evaluated using sine wave grating charts of FACT with five spatial frequencies in photopic conditions (85 candela/m2) in far vision and without glare mode. The spatial frequencies consisted of 1.5 cycle/per degree (cpd) (threshold range 0.045-2.00), 3 cpd (threshold range 0.70-2.20), 6 cpd (threshold range 0.78-2.26), 12 cpd (threshold range 0.60-2.08) and 18 cpd (threshold range 0.30-1.81). There are nine gradually blurred gratings available in each spatial frequency. While testing CS, the participants were asked to describe the position (right, up, or left) of nine gratings in each row that corresponds to each spatial frequency. The true position of the last grating that could be identified by the participant in a tested row was accepted as the CS score of the tested spatial frequency. The CS score for each spatial frequency was then transformed to logarithmic value.

### Optical Coherence Tomography Imaging

The 512x128 macular cube and 200x200 optic disc cube protocols of Cirrus HD-OCT were used to obtain macular scan and optic nerve head scan, respectively, for the purpose of measuring the central foveal thickness (CFT), GCIPL thickness, and peripapillary RNFL thickness. The average, minimum and sectoral (superotemporal, superior, superonasal, inferonasal, inferior, inferotemporal) GCIPL thicknesses of each participant were measured from ganglion cell analysis algorithm. The average and sectoral (superior, inferior, nasal and temporal) thicknesses of peripapillary RNFL of each participant were also measured.

### Statistical Analyses

Statistical analyses were performed using SPSS software version 15. The variables were investigated using visual (histograms, probability plots) and analytical methods (Kolmogorov-Smirnov/Shapiro-Wilk test) to determine whether or not they were normally distributed. Descriptive analyses were presented using means and standard deviations for all variables. Since the average, minimum and sectoral GCIPL thickness, the average and sectoral RNFL thickness and SE value of participants were normally distributed, the Student’s t-test was used to compare these parameters between the insulin resistant and control groups. The Mann-Whitney U test was used for intergroup comparisons of spatial frequency CS scores, plasma insulin level, BMI value, and HOMA-IR value because these parameters did not show normal data distribution. Additionally, the associations between average GCIPL thickness and plasma insulin level, fasting plasma glucose level (FPGL), BMI value and HOMA-IR value were evaluated using the Spearman test. A p-value of less than 0.05 was considered to show a statistically significant result.

## RESULTS

**Figure 1:**
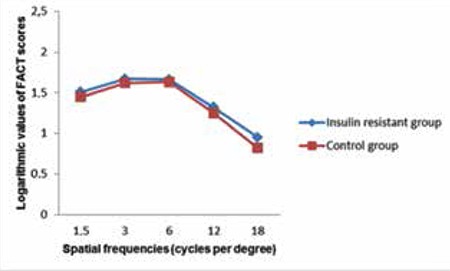
The mean functional acuity contrast test scores in terms of logarithmic values at all spatial frequencies in the insulin resistant group and control group FACT: Functional acuity contrast test

**Figure 2:**
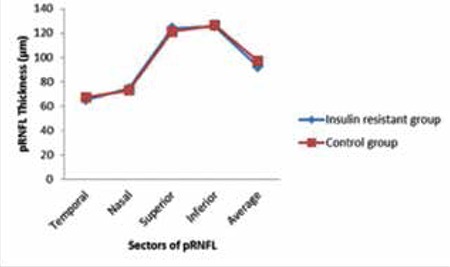
The mean average and sectoral (temporal, nasal, superior, inferior) thicknesses of peripapillary retinal nerve fiber layer in the insulin resistant group and control group pRNFL: Peripapillary retinal nerve fiber layer

Twenty-five eyes of 25 patients with insulin resistance (insulin resistant group), and 25 eyes of 25 healthy subjects (control group) were included in this study. There was no significant difference in terms of age, sex and mean SE value between the insulin resistant group and control group. However, in comparison with the control group, the insulin resistant group showed significantly higher values in other parameters such as mean plasma insulin level, mean FGL, mean BMI value, and mean HOMA-IR value (Table 1). The mean values and statistical comparisons of average, minimum and sectoral GCIPL thicknesses between insulin resistant group and control group are shown in Table 2. The mean average GCIPL thickness was found to be significantly thinner in the insulin resistant group than in the control group (83.6±4.7 µm vs. 86.7±3.7 µm, respectively, p=0.01). Among the sectoral GCIPL parameters, only the mean inferotemporal thickness was significantly thinner in the insulin resistant group compared to control group (83.0±6.0 µm vs. 86.7±4.6 µm, respectively, p=0.02). The mean CFT of the insulin resistant group and control group was 243±19 µm and 249±16 µm, respectively (p=0.4). Spearman’s correlation test showed that there were negative correlations between average GCIPL thickness and insulin plasma level, BMI value and HOMA-IR value (Table 3). The mean logarithmic values of FACT scores measured in each spatial frequency of the insulin resistant group and control group were in normal range (1.51±0.19 vs 1.45±0.16, p=0.2 at 1.5 cpd; 1.67±0.25 vs 1.62±0.20, p=0.4 at 3 cpd; 1.66±0.26 vs 1.63±0.18, p=0.5 at 6 cpd; 1.32±0.25 vs 1.25±0.16, p=0.2 at 12 cpd; and 0.95±0.33 vs 0.82±0.29, p=0.1 at 18 cpd). As can also be seen in Figure 1, there were no statistically significant differences between the insulin resistant group and control group in low (1.5 cpd), middle (3 and 6 cpd) or high (12 and 18 cpd) spatial frequencies of the CS test. The mean average and sectoral RNFL thicknesses were similar between two groups (Figure 2).

## DISCUSSION

The exact cause of the retinal neurodegeneration seen in diabetic patients has not been determined yet, but strong evidence from animal studies has demonstrated the significant role of apoptosis in the retinal cell death of diabetic patients. In a histopathological examination, a significant number of apoptotic RGCs, as well as pronounced reduction in the thickness of both inner-plexiform and inner nuclear layers was shown in streptozotocin-induced diabetic rat retina.^20^ The increased susceptibility of RGCs to apoptosis in diabetics was also confirmed by demonstrating the increased number of both apoptosis markers such as TUNEL positive cells and caspase-3 positive cells in the RGC layer of streptozotocin-induced diabetic mice.^21^ Aside from animal studies, RGC loss due to diabetes was also shown in a number of postmortem human studies.^22^

Increased apoptotic damage of RGCs in diabetic patients can be due to impaired retinal insulin receptor signaling, which works on the phosphatidylinositol 3-kinase (PI3-K)/Akt signaling pathway. It has been shown that both insulin and insulin-like growth factor-1 (IGF-1) can act as a trophic factors for the survival of retinal neurons including RGCs through a PI3-K/Akt signaling pathway.^23,24^ In this pathway, a conformational change in the retinal insulin receptor after insulin stimulation is thought to cause a series of phosphorylations in which phosphorylated PI3-K activates Akt, then phosphorylated Akt inhibits apoptosis by phosphorylating caspase-9, which is the head of proteolytic cascade.^25^ On the other hand, it has been shown that insulin and IGF-1 need to use insulin receptor substance (IRS) as an integrating factor in order to properly transmit the survival signal to the PI3-K/Akt signaling pathway.^26^ The deficiency of IRS-2 in mice was revealed to induce loss of RGC and photoreceptors, which is associated with decreased Akt activation and increased caspase-3 activation.^27^ Therefore, deterioration in the retinal insulin/IGF receptor signaling pathway or deficiency in its intermediary components such as IRS has been associated with neurodegeneration and retinopathy.^28^

Although the association between diabetes/retinal cell apoptosis and the onset time of diabetic retinal neurodegeneration has been well documented, less is known about whether RGC loss also occurs in the period of insulin resistance (in other words, prior to the development of diabetes). In the pathogenesis of insulin resistance, suppression of cytokine signaling 1 (SOCS-1) and SOCS-3 has been suggested to cause an impairment in insulin signaling in the liver through inhibiting the activity of IRS-1 and IRS-2.^29^ Furthermore, persistent expression of SCOS-3 has also been implicated in the development of retinal insulin resistance by leading to diminished activity of IRS-2 in rats.^30^ Taken together, it is conceivable to think that patients with insulin resistance may suffer from decreased visual function as a result of reduced RGC number. Dosso et al.^31^ demonstrated the impaired CS function in both insulin resistant obese patients and diabetic patients without retinopathy, and they associated this result with the possible involvement of RGCs.

Despite the numerous studies which have exhibited the damage of RGCs in diabetic patients, to our knowledge there are no published studies investigating the status of RGCs in insulin resistant patients. Since increased apoptosis has been shown to cause thinning of the RGC layer or RNFL,^32^ in the present study we compared GCIPL and RNFL thicknesses in insulin resistant patients with those measured in healthy subjects. We determined that the mean average GCIPL thickness in insulin resistant patients was significantly less in comparison with the healthy subjects. Because the abundance of both insulin and its receptors has been shown in the retina, especially in the inner plexiform layer,^33^ the decrease in the average GCIPL thickness of insulin resistant patients may be consistent with the possibly increased apoptosis in RGCs. In previous studies, decreased RGC layer thickness was shown in patients with either type 1 or type 2 diabetes.^13,14^ However, the correlation between RGC layer thickness and plasma insulin level, which varies according to diabetes type, was not assessed in these studies.

In the present study, we evaluated the correlation between average GCIPL thickness and the parameters of insulin resistance such as plasma insulin level, BMI value, and HOMA-IR value. We detected statistically significant negative correlations between mean average GCIPL thickness and all of the insulin resistance parameters. However, increased BMI had a more pronounced effect on average GCIPL thickness compared to plasma insulin level and HOMA-IR value. This result may be due to insufficient perfusion of the RGCs, because increased levels of vasoconstrictor molecules (endothelin-1 and angiotensin-2), which can lead to impaired perfusion, have been associated with higher BMI.^34,35^ Moreover, detection of narrowed retinal arterioles in patients with higher BMI may indicate possible deterioration in the microvascular circulation of inner retinal layers.^36^ Although mean FPGL was in the normal range in both groups, it was significantly higher in the insulin resistant patients. Nevertheless, we did not detect any correlation between mean FPGL and GCIPL thickness. The evaluation of this correlation may have importance in terms of ruling out the possible apoptotic effect of increased blood glucose level on RGCs, and therefore demonstrating the significant effect of impaired insulin action on retinal neurodegeneration, since both hyperglycemia and deficiency in insulin action have been reported to be involved in apoptotic cell damage of the diabetic mouse retina.^37^

It has been previously suggested that some of the RGCs participate in the parvocellular (P cells) system, which is thought to function in high-contrast, high-spatial resolution, while other RGCs participate in the magnocellular (M cells) system, which is thought to function in low-spatial contrast.^38^ Therefore, in this study we performed the CS test at low (1.5 cpd), middle (3 cpd and 6 cpd), and high (12 cpd and 18 cpd) spatial frequencies in order to assess the function of M cells and P cells and thereby evaluate RGC function. We found similar scores between the insulin resistant and control groups at all spatial frequencies. The preservation of CS function in insulin resistant patients may be due to mild RGC loss; considering the findings of Zhang et al.,^39^ it may be estimated that there was a 4% difference in the number of macular RGCs between the two groups in our study. However, it has been reported that functional visual loss occurs in cases with damage to 20% to 40% of RGCs.^40^

### Study Limitations

Despite a number of studies suggesting the relationship between axial length (AL) of the eye and thickness of GCIPL,41 we could not assess the AL of the participants during this study due to the absence of an optical biometer in our clinic. However, we ensured the participants in our study had comparable, low SE values. Apart from the small sample size, we consider the absence of AL measurement a major limitation of this study.

## CONCLUSION

Although the decreases observed in average and inferotemporal GCIPL thicknesses were not found to cause loss of CS function in patients with insulin resistance, it may provide evidence that retinal neurodegeneration likely begins in the insulin resistance stage.

### Ethics

Ethics Committee Approval: The study were approved by the Çanakkale Onsekiz Mart University of Local Ethics Committee, Informed Consent: It was taken.

Peer-review: Externally peer-reviewed.

## Figures and Tables

**Table 1:**
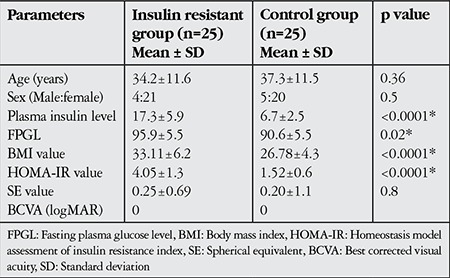
Demographics of insulin resistant group and control group

**Table 2:**
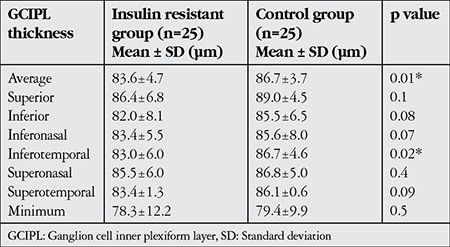
The mean values and statistically comparisons of average, minimum and sectoral thicknesses of ganglion cell inner plexiform layer between insulin resistant group and control group

**Table 3:**
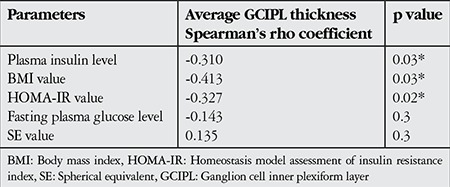
Spearman’s rank correlation coefficients between average ganglion cell inner plexiform layer thickness and insulin resistance parameters and spherical equivalent value
